# A description of village chicken production systems and prevalence of gastrointestinal parasites: Case studies in Limpopo and KwaZulu-Natal provinces of South Africa

**DOI:** 10.4102/ojvr.v83i1.968

**Published:** 2016-05-12

**Authors:** Dikeledi P. Malatji, Anna M. Tsotetsi, Este van Marle-Koster, Farai C. Muchadeyi

**Affiliations:** 1 Biotechnology Platform, Agricultural Research Council, South Africa; 2 Department of Wildlife and Animal Science, University of Pretoria, South Africa; 3 Parasites, Vectors and Vector-borne Diseases Program, Agricultural Research Council, South Africa; 4 Department of Zoology and Entomology, University of Free State, Qwaqwa Campus, South Africa

## Abstract

The majority of rural households in developing countries own village chickens that are reared under traditional scavenging systems with few inputs and exposure to various parasitic infestations. Understanding of the village chicken farming system and its influence on helminth infestation is a prerequisite for optimal prevention and control strategies. This study investigated the village chicken production system and associated gastrointestinal parasites in 87 households from Limpopo (*n* = 39) and KwaZulu-Natal (*n* = 48) provinces of South Africa. A total of 191 village chicken faecal samples and 145 intestines were collected to determine the prevalence of gastrointestinal parasites in villages of Limpopo and KwaZulu-Natal provinces, respectively. The faecal floatation analysis of samples from Limpopo and KwaZulu-Natal provinces indicated infestations by *Ascaridia galli* (18.77%), *Heterakis gallinarum* (15.56%) and *Capillaria* spp. (4.00%); tapeworms *Choanotaenia infundibulum* (2.10%) and *Raillietina cesticillus* (6.00%) and *Eimeria* spp. (29.46%). Mixed infestations were observed in five (4.90%) samples from Limpopo province and in only four (4.49%) from KwaZulu-Natal province, of which 1.12% were a mixture of *C. infundibulum* and *Eimeria* spp. and 3.37% a combination of *H. gallinarum* and *Eimeria* spp. In Limpopo, 2.94% of the chickens were positive for *H. gallinarum* and *Eimeria* spp., whilst 0.98% had *A. galli* and *Capillaria* spp. infestations. Further investigation is needed to understand the impact of gastrointestinal parasites on village chicken health and production and develop appropriate intervention and control strategies feasible for small-holder farmers.

## Introduction

Village chickens are poultry mostly owned by village communities in rural areas of Africa (Thekisoe, Mbati & Bisschop 2004) and other developing countries (Muchadeyi *et al*. 2004; Muhiye 2007). They play a vital role through their contribution to the socio-economic and cultural lives of small-holder farmers (Nyoni & Masika 2012; Van Marle-Köster *et al*. 2008). Village chickens can be used as tokens of appreciation for services rendered and are often given to visitors as gifts (Kusina & Kusina 1999). Their role in national economies is through improved nutritional status and income of many small-holder farmers as well as landless and marginalised communities (Muchadeyi *et al*. 2004; Tarwireyi & Fanadzo 2013).

A majority of chicken populations in Africa are kept under traditional scavenging systems (McAinsh *et al*. 2004; Mtileni *et al*. 2009) that are often characterised by low productivity and high mortality. Village chickens are left to scavenge to meet their nutritional needs (Muchadeyi *et al*. 2004; Mwale & Masika 2009), which predisposes them to predators (Kusina & Kusina 1999; Pedersen 2002), diseases and parasites (Swatson *et al*. 2003) that coexist in the scavenging environment. Parasite infestation contributes to poor production and can cause mortality in severe cases (Soulsby 1982).

Gastrointestinal parasites are the most prevalent parasites affecting the productivity of village chickens (Mwale & Masika 2011). Their prevalence in village chickens has been studied in different countries, and to a lesser extent in South Africa (Mukaratirwa & Khumalo 2010; Mwale & Masika 2011). Different species of endo-parasites have been identified (Muhairwa *et al*. 2007; Permin *et al*. 2002), including endo-parasites such as *Eimeria* spp. and helminths (Norton & Ruff 2003). Parasitic infestation rates have been shown to differ amongst different production systems (Permin *et al*. 1999) because of variations in environmental and management factors. Improved poultry management practices are responsible for reduction in the incidence of parasitic infestations (Puttalakshmamma *et al*. 2008). Kaufmann (2011) indicated that village chickens do not only harbour a wide spectrum of helminths but are also associated with relatively high intensity of infestations compared to commercial chickens with more frequent incidences in free range than intensive systems.

The small-holder farming sector of South Africa is similar to those in other African and developing countries that are characterised by low production inputs, exposure of chickens to diseases and parasites and compromised biosecurity and veterinary interventions (Acavomic *et al*. 2005). Therefore, the aim of the study was to describe typical village chicken production systems in selected village farms in Limpopo and KwaZulu-Natal provinces of South Africa and to determine the prevalence of gastrointestinal parasites in these communal low-input farming systems. Such information is considered important for the development and implementation of effective control programmes.

## Materials and methods

### Study sites and animal populations

**FIGURE 1:**
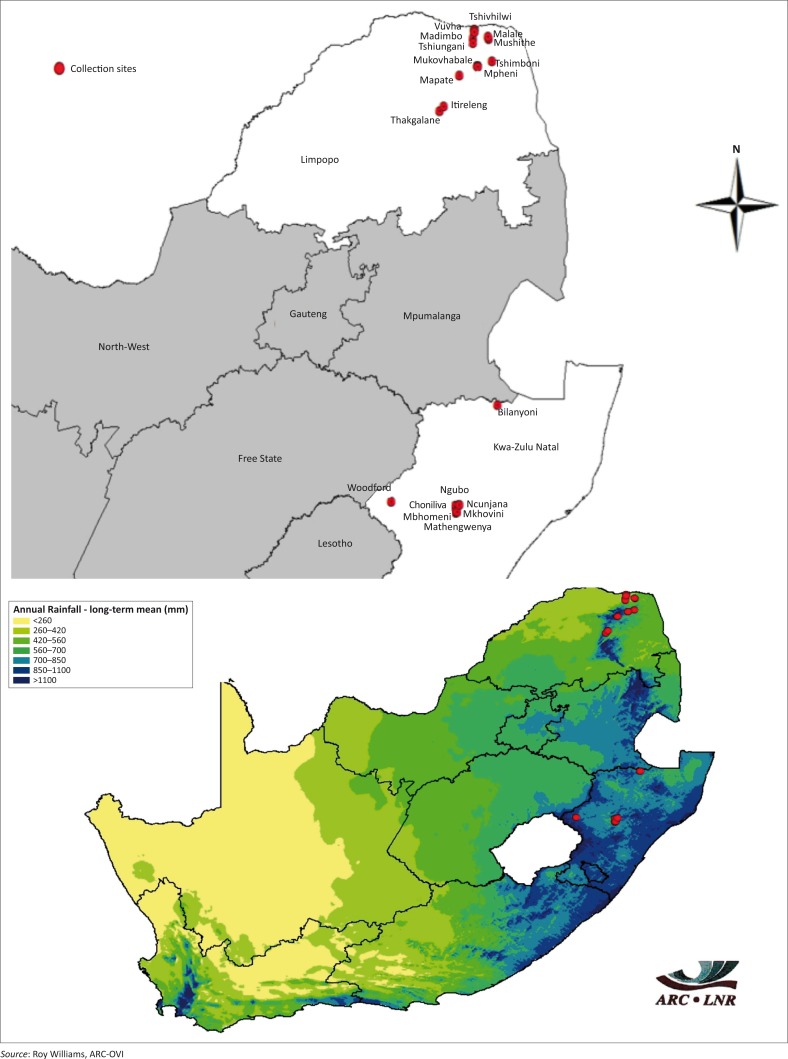
Map of South Africa showing sampled villages (red dots) in Limpopo and KwaZulu-Natal provinces.

This study was conducted in 18 villages in two agro-ecological zones of the Limpopo and KwaZulu-Natal provinces of South Africa (Figure 1). These provinces were targeted based on the existence of free-range village chicken production and the contrasting environmental conditions between them. Mopani District in Limpopo province is situated within the subtropical zone. It can be very hot in summer, reaching maximum temperatures of 38 °C. Winters are mild during the day and cold during the nights. Vhembe District in Limpopo province experiences a hot semi-arid climate with hot temperatures most of the year. Average annual precipitation amounts to 372 mm with extremely dry winters. uThukela District municipality in KwaZulu-Natal province covers an area of approximately 11 500 km². It experiences heavy snow on the mountains in winter. It is located in the western boundary of KwaZulu-Natal province. uMzinyathi District has a temperate climate. Frost occurs only in parts of uMzinyathi in winter. Rainfall varies from more than 800 mm in Endumeni and Umvoti to less than 400 mm in parts of Msinga. Eighty-seven households were randomly selected from villages of the two provinces from November 2012 to February 2013. Information was gathered from veterinary extension officers on chicken ownership in the two provinces. Households in each village were then selected on the basis of the availability of free-ranging chickens and the willingness of the chicken owners to participate in the study.

### Questionnaire survey

One-on-one interviews with farmers were conducted with assistance from the agricultural extension officers from the Department of Agriculture in Vhembe District, Limpopo province, and from community extension personnel of the Mdantsane Non-Governmental Organization (NGO) in Tugela Ferry in KwaZulu-Natal province between November 2012 and February 2013. Standardised questionnaires were administered to the 87 randomly selected households from villages of Limpopo (*n* = 39) and KwaZulu-Natal (*n* = 48) provinces. The questionnaires were semi-structured with both closed and open-ended questions that were designed to capture information on the village chicken production systems with emphasis on the different livestock species kept by farmers, roles of village chickens, chicken nutrition, housing and health management and access of farmers to agricultural extension services. Information was also captured during these interviews on any chicken disease or clinical signs observed by farmers in their flocks. Farmers were then asked to rank the prevalent diseases or clinical signs in order of importance. Chicken production parameters that included number of eggs/clutch/hen, number of egg clutches per hen per annum and number of eggs that hatched per clutch were recorded. Farmers in both provinces did not keep farm records, and therefore, data collected were based on farmer recall.

### Sample collection and parasite identification

A total of 191 free-ranging village chicken faecal samples were collected from individual village chickens from the same households interviewed in Limpopo (102 faecal samples from 34 households) and KwaZulu-Natal provinces (89 faecal samples from 47 households). Freshly voided faecal samples were collected by following chickens and monitoring them within the household, chicken pens and the surroundings. After collection, faecal samples were stored at 4 °C until further analysis to prevent the eggs of parasites from hatching. The modified quantitative McMaster floatation technique was used to examine faecal samples (MAFF 1986).

In addition to the faecal samples, 145 live mature chickens were purchased from the same villages in Limpopo (*n* = 99) and KwaZulu-Natal (*n* = 46). These chickens were slaughtered and the gastrointestinal tracts were removed from the proventriculus to the cloaca after which each region was cut open by dissection following the World Association for the Advancement of Veterinary Parasitology guidelines for evaluating the effectiveness of anthelmintics in chickens and turkeys (Yazwinski *et al*., 2003). All gastrointestinal parasites visible to the naked eye were recovered from the GIT of the chickens using thumb forceps, washed and stored in 70% ethanol at ambient temperature awaiting parasite identification. Identification of each parasite was carried out based on the morphological parameters using the helminthological keys (Norton & Ruff 2003; Soulsby 1982).

### Data analysis

Statistical Analysis System was used to analyse questionnaire-derived variables such as flock size, flock composition, diseases, disease clinical signs, internal parasites, external parasites, vaccination, treatment and access to veterinary services. Descriptive statistics using Generalized Linear Model procedures, SURVEYMEANS and SURVEYFREQ procedures were computed and presented as tables and graphs. The level of significance was considered at *p* ≤ 0.05. For each province, the number of eggs/clutch/hen, number of egg clutches per hen per annum and number of eggs that hatched per clutch were averaged into mean clutch size, mean number of clutches and average hatchability, respectively.

The prevalence of each recovered and identified GIT parasite was calculated as the number of chickens infested with that particular parasite species, divided by the total number of chickens sampled (Thrusfield 1990). The prevalence of the gastrointestinal parasites was calculated per province using Statistical Analysis System (SAS 2003). The mean intensity was determined by dividing the total number of recovered parasites of a particular species by the number of chickens infested with that parasite (Bush *et al*. 1997). Abundance was calculated by dividing the number of parasites of a particular species by the total number of chickens examined (Bush *et al*. 1997).

## Results

### Village chicken flock sizes and composition

A total of 858 and 1351 village chickens were reported by the village farmers in Limpopo and KwaZulu-Natal provinces, respectively (Table 1). The least square means ± standard error of flock size per household was 22.03 ± 2.85 for Limpopo province and 28.40 ± 2.57 for KwaZulu-Natal province. Flock sizes varied between farms within provinces and about 44.68% of the farmers from both provinces had between 10 and 30 chickens. Twenty-seven percent of the farms owned 30–50 chickens and 8.5% owned 50–100 chickens. Only 6.3% of the interviewed farmers had fewer than 10 chickens. Cock:hen ratios of 1:3 and 1:4 were observed in Limpopo and KwaZulu-Natal provinces, respectively.

**TABLE 1:** Least squares means ± standard error of the flock sizes and composition in Limpopo and KwaZulu-Natal provinces of South Africa.

Category of chickens	Limpopo Province†		KwaZulu-Natal Province‡	
	
	Total number of chickens	LSM ± SE	Total number of chickens	LSM ± SE
Total flock size	858	22.03 ± 2.85	1351	28.40 ± 2.57
Hens	374	9.59 ± 1.51	638	13.50 ± 1.36
Cocks	125	3.23 ± 0.44	152	3.19 ± 0.40
Chicks	359	9.21 ± 1.08	561	11.71 ± 1.62

† *N* = 39 households;

‡ *N* = 48 households.

LSM, least squares means; SE, standard error.

### Role of village chickens

Village chickens were predominantly kept for providing meat for household consumption (51% and 37%), sale (15% and 2%) and a combination of meat and sale (25.6% and 29.2%) and for meat and eggs (5.1% and 4.2%) in Limpopo and KwaZulu-Natal provinces, respectively, as well as other functions including investment and rituals (Table 2). Most farmers indicated that they preferred village chicken meat as a supplement to their nutritional diets because it is tastier compared to intensively raised commercial breeds. Families also benefited indirectly from rearing village chickens through the use of manure for vegetable gardens. Farmers also indicated that chickens control weeds and insect pests by foraging.

**TABLE 2:** The percentage of farmers reporting the different roles of village chickens in the selected villages of Limpopo and KwaZulu-Natal province of South Africa.

Use of chicken	Province	

	Limpopo (*N* = 39)†	KwaZulu-Natal (*N* = 48)†
Meat	51.28	37.5
Selling	15.38	2.08
Investment	0	2.08
Meat and selling	25.64	29.17
Meat and eggs	5.13	4.17
Meat and investment	0	12.5
Meat, selling and rituals	2.56	10.42

† Number of households.

### Village chicken production systems

**FIGURE 2:**
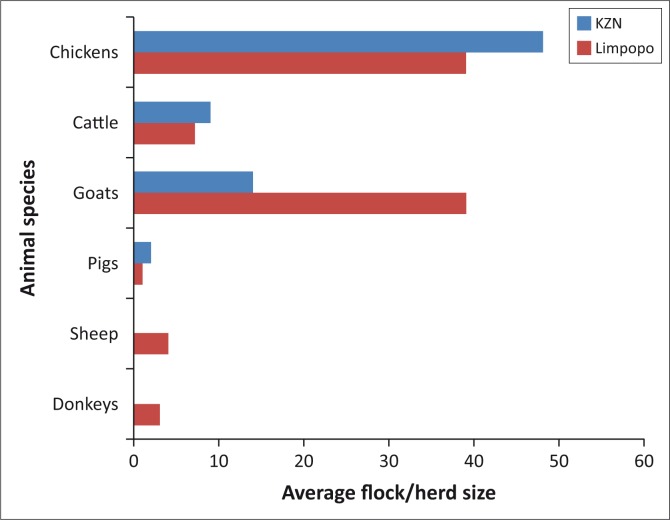
Livestock owned by farmers in the study areas of Limpopo and KwaZulu-Natal provinces.

In addition to rearing village chickens, farmers in both provinces kept goats and cattle, as well as sheep, pigs and donkeys (Figure 2). Chickens were the predominant species in both provinces, followed by cattle and goats. In Limpopo, 94.9% farmers provided chicken housing at night. Chicken structures in this province were made from locally available materials such paper boxes, scrap wood from discarded household furniture and wooden poles. Farmers in KwaZulu-Natal province did not provide housing for their chickens, which find shelter in trees and homestead kitchens. Village chickens in both provinces were left to scavenge around the homestead and surroundings for food and water during daytime.

The typical diet for the village chickens consisted of locusts and other insects, earthworms, grass, discarded food and vegetables. Scavenging was supplemented with kitchen leftovers (16.72% and 4.59%), maize grain (28.73% and 52.26%), commercial feed (16.68% and 1.15%) and a combination of maize grain and husks and other leftovers (30.64% and 1.15%) in Limpopo and KwaZulu-Natal provinces, respectively. Because of the nature of the production system practised, farmers did not have a well-organised and controlled chicken-breeding programme. Broody hens would naturally incubate their own eggs, and none of the farmers practised artificial incubation. Hens laid an average of three clutches per year with an average of 12.08 and 13.64 eggs per clutch in Limpopo and KwaZulu-Natal provinces, respectively. The average hatchability rate was 76.32% and 78.31% for Limpopo and KwaZulu-Natal provinces, respectively. Farmers increased their flocks by sharing chickens (6.89% and 22.98%) amongst friends and relatives in Limpopo and KwaZulu-Natal provinces, respectively.

Over 71.80% of the farmers reported that Newcastle disease was the most important constraint that caused chicken mortality in both the provinces. Of the farms in Limpopo and KwaZulu-Natal provinces, 23% and 49%, respectively, were affected by Newcastle disease. Other diseases of importance were fowlpox and infectious bursal disease. The most predominant chicken disease clinical signs observed by farmers in descending order of importance in both provinces were generalised weakness (3.44% and 11.49), swollen eyes (6.89% and 6.10%), diarrhoea (8.05% and 0.00%) and coughing (5.75% and 1.15%). Other clinical signs included chickens being unable to walk (3.44% and 2.29%) and salivation (1.15% and 0.00%) in Limpopo and KwaZulu-Natal provinces, respectively. Of all farmers interviewed during this study, only 6.89% in Limpopo and 29.32% in KwaZulu-Natal had observed gastrointestinal parasites in chicken droppings. A proportion of 11.49% and 66.23% of the farmers observed the presence of external parasites on their chickens in Limpopo and KwaZulu-Natal provinces, respectively.

### Vaccination, treatment and access to veterinary interventions

Ninety-four percent of the farmers interviewed in KwaZulu-Natal province used NOBILIS^®^ ND CLONE 30 vaccine that was provided by Mdantsane NGO to vaccinate their chickens against Newcastle disease. Other farmers in KwaZulu-Natal province used this vaccine to treat their chickens against diseases as they only administered it when chickens had already developed clinical signs. The frequency of vaccination varied amongst farms depending on the knowledge they had and also on the clinical signs observed in the chicken flocks. Most of the farmers (52.10%) administered ND clone vaccine once every 2 months, whereas 14.58% used it every month. Only 12.50% of the farmers used it once per annum. A total of 10.42% of the farmers used vaccination randomly or when the need arose. Farmers in Limpopo province did not use any vaccination to manage the health of their animals.

In Limpopo province, 17 (43.59%) of the farmers interviewed used ethno-veterinary medicine such as aloe, garlic and hot chilli pepper for treating chicken diseases and parasites. Farmers indicated that they had more faith in the use of piperazine, Jeyes fluid and laundry powders in that order. None of the farmers interviewed in KwaZulu-Natal province used ethno-veterinary medicine, and none of the farmers from either province used antibiotics and/or anthelmintic to treat diseases and parasites. It was also observed that all farmers (100.00%) in the surveyed villages of Limpopo province had never received extension support from the Department of Agriculture. In KwaZulu-Natal, a total of 47 farmers (97.92%) received veterinary interventions provided by the Mdantsane NGO.

### Prevalence of gastrointestinal parasites

Fourteen (15.73%) and 43 (42.16%) chicken faecal samples were positive for gastrointestinal parasites in KwaZulu-Natal and Limpopo provinces, respectively. Six different parasite species that included nematodes *Ascaridia galli, Heterakis gallinarum* and *Capillaria* spp., tapeworms *Choanotaenia infundibulum* and *Raillietina cesticillus* and protozoa (Coccidia) *Eimeria* spp. were identified in both KwaZulu-Natal and Limpopo provinces (Table 3). Five (4.90%) samples from Limpopo and four (4.49%) from KwaZulu-Natal had mixed infestations in which the animal was infested by more than one parasitic species. *Ascaridia galli* was the most prevalent parasite at 17.65% in Limpopo followed by *Eimeria* spp. (13.73%), *H. gallinarum* (8.82%), *R. cesticillus* (4.90%), *Capillaria* spp. (2.94%) and *C. infundibulum* at 1.00%. The results from KwaZulu-Natal province indicated a lower prevalence of gastrointestinal parasites except for *Eimeria* spp. at 15.6% (Table 3). The prevalence of *H. gallinarum* was 6.74% and that of *A. galli, Capillaria* spp. and *R. cesticillus* was 1.10% in KwaZulu-Natal province. No trematodes were observed in either province. In this study, variation in the prevalence of these parasites was observed between the two different provinces, although they were not statistically significant.

**TABLE 3:** Prevalence (%), least squares means (LSM ± SE) and range of gastrointestinal parasite species from faecal samples from village chickens of Limpopo and KwaZulu-Natal provinces of South Africa.

Gastro-intestinal parasite	Parasite type	Limpopo Province (*N* = 102 faecal samples)			KwaZulu-Natal Province (*N* = 89 faecal samples)		
	
		Prevalence	LSM ± SE	Range	Prevalence	LSM ± SE	Range
Nematodes	*Ascaridia galli*	17.65	0.18 ± 0.04a	0–2600	1.12	0.011 ± 0.011b	0–50
	*Heterakis gallinarum*	8.82	0.09 ± 0.03	0–1360	6.74	0.07 ± 0.03	0–250
	*Capillaria* spp.	2.94	0.03 ± 0.02	0–200	0	0	0
Tapeworms	*Choanotaenia infundibulum*	0.98	0.01 ± 0.01	0–150	1.12	0.01 ± 0.01	0–50
	*Raillietina cesticillus*	4.9	0.05 ± 0.02a	0–200	0	0b	0
Protozoa	*Eimeria* spp.	13.73	0.14 ± 0.03	0–1500	15.73	0.16 ± 0.04	0–750

Means with different superscript alphabets in the same row are significantly different (*p* < 0.05).

Figures in the range columns represent the range for the actual values (untransformed data) of eggs per gram of faeces.

LSM, least squares means; SE, standard error.

Twenty-nine (64.44%) of the 45 chickens slaughtered in KwaZulu-Natal were positive for either one or two adult parasite species. Mixed infestations of *A. galli* and tapeworm were observed in 27.59% of the infested animals, whilst the remaining 72.41% were positive for only one parasite species. In Limpopo province, 36 (36.36%) of the 99 slaughtered chickens were positive for at least one parasite species. Tapeworms were the most prevalent parasites at 75.00%, followed by *A. galli* at 52.78% and *H. gallinarum* at 8.33%. Mixed infestations were observed in 15 (41.67%) of the intestines, 6.67% of which were with *A. galli* and *H. gallinarum* and 93.33% were with *A. galli* and tapeworms. The tapeworms were not identified to species.

A total of 201 parasites from 36 positive chickens and 228 from 29 positive chickens were observed in Limpopo and KwaZulu-Natal provinces, respectively (Table 4). The average number of worms recovered per animal was 5.9 ± 5.43 in Limpopo and 7 ± 12.39 in KwaZulu-Natal province. The chickens that harboured more worms were observed in KwaZulu-Natal province, where a total of 150 *A. galli* parasites were recovered from a single chicken. However, the chicken that carried these 150 parasites was not included in the statistical analysis as it was considered an outlier that was going to inflate the average number of worms per animal. In KwaZulu-Natal province, the average intensity of infestation was highest for *A. galli*, with an average worm count of 7.2 ± 13.76 per chicken. The highest mean abundance of infestation was seen in *A. galli* (3.61 ± 10.28 worms per chicken) followed by tapeworm (1.35 ± 2.13 worms per chicken) (Table 5). In≈Limpopo, the intensity was high for *H. gallinarum* at 8.67 ± 2.32 worms per chicken. Mean abundance of infestation was high at 1.11 ± 3.27 worms per chicken for tapeworms.

**TABLE 4:** Total worm count and mean worm intensity for slaughtered free-range chickens from the selected villages in Limpopo and KwaZulu-Natal provinces of South Africa.

Gastro-intestinal parasite	Parasite species	Total worm count (mean worm intensity per bird)					

		Limpopo Province		KwaZulu-Natal Province		Total per species	
		
		Worm count	Mean worm intensity	Worm count	Mean worm intensity	Worm count	Mean worm intensity
Nematodes	*Ascaridia galli*	65	3.42	166	7.2	231	8.71
	*Heterakis gallinarum*	26	8.67	0	0	26	-
Tapeworms†	Tapeworm	110	4.07	62	4.13	172	-

**Number of chickens**		**36**		**29**		**65**	
**Total worm count per province**		**201**		**228**		**-**	

† Tapeworms were not identified to the species level.

**TABLE 5:** Mean worm abundance ± standard deviation from the intestines of slaughtered chickens from Limpopo and KwaZulu-Natal provinces of South Africa.

Gastro-intestinal parasite	Parasite species	Province (Abundance ± SD)	

		Limpopo†	KwaZulu-Natal‡
Nematodes	*Ascaridia galli*	0.66 ± 1.65	3.61 ± 10.28
	*Heterakis gallinarum*	0.25 ± 2.05	0
Tapeworms	Tapeworm§	1.11 ± 3.27	1.35 ± 2.13

† *N* = 99 chickens;

‡ *N* = 46 chickens;

§ The tapeworms could not be differentiated at the species level.

SD, standard deviation.

## Discussion

This study described the village chicken production systems and associated parasite infestations in two provinces of South Africa. Village chicken production in South Africa is similar to most small-holder farming systems where the chickens are exposed to the harsh environmental and production challenges coupled with farmers having limited resources to manage their flocks (Acavomic *et al*. 2005). Therefore, an understanding of the dynamics and challenges of helminth infestation in the context of the production systems is crucial to the development and implementation of effective parasite control strategies. This study sampled from villages of Limpopo and KwaZulu-Natal provinces of South Africa, which are similar to villages in most African and other developing countries.

The proportion of hens was high in both provinces, which is in contrast to other studies, which observed village chicken flocks being dominated by chicks (Maphosa *et al*. 2004; Muchadeyi *et al*. 2004). It was expected that high rates of helminth infestations would be observed with increased flock sizes because of the increased number of animals per unit area that leads to larger amounts of faeces deposited on the ground and possibly increased infectivity per unit area (Permin & Nansen 1998). The relatively low levels of helminth infestations observed regardless of flock numbers in KwaZulu-Natal could be attributed to the extensive management of these chickens and the hot and dry conditions during sampling, which negatively affect the development of parasite eggs into infective stages and their survival in the environment (Permin & Nansen 1998). Farmers in both provinces practised mixed livestock farming and owned goats, cattle, sheep and pigs in addition to chickens. However, the presence of other animal species in mixed livestock farming systems can expose and increase the risk of parasite infestation in village chickens as other species could act as carriers of certain parasites (Permin & Nansen 1998).

The majority of chickens in this study scavenged for their feed, an observation that was consistent with other studies (Muchadeyi *et al*. 2007; Mwale & Masika 2009). Whilst this is a viable option for resource-limited farmers, scavenging for feed results in poor quality nutrition and also exposes the chickens to predation, diseases and parasites (Acamovic *et al*. 2005). Scavenging chickens are also exposed to the open air and environment and have greater contact with host organisms such as insects and the earthworm where they can be infested. Insects and earthworms are intermediate hosts that may indirectly transmit the parasite eggs and infective stage of nematodes to chickens on consumption (Butcher & Miles 2009). The prevalence of parasites in Limpopo province would probably be lower if the chicken housing was well managed with cleaning, removal of the droppings and regular application of disinfectants (Kusina & Kusina 1999; Pedersen 2002). Farmers in the surveyed villages were also observed to exchange animals with their neighbours and nearby community members. According to the conventional commercial chicken management practices (Kitalyi 1998), new and introduced stock needs to be isolated and monitored for a certain period of time so that the farmer does not introduce diseases and parasites to the farms. Such practices, although challenging for small-holder communal farmers, can help in reducing the introduction and spread of new pathogens between neighbouring farms and/or communities in village chicken and other livestock production systems.

The reported diseases and clinical signs were merely based on farmer’s perceptions and their limited knowledge of clinical signs. The majority of the farmers did not have access to veterinary extension services, whilst those in KwaZulu-Natal province received extension services from a NGO working in that area. The administration of vaccines randomly without clear indications for the need for vaccinations and proper procedures being followed could result in poor response to vaccines.

The prevalence of *A. galli* in both provinces was higher than the 10.0% – 14.5% reported in Kenya (Irungu, Kimani & Kisia 2004). However, the prevalence of nematode infestations (combination of *A. galli* and *H. gallinarum* prevalence) was low compared with those observed in Zambian villages, which revealed that 28.8% and 32.8% of the chickens were infested with *A. galli* and *H. gallinarum*, respectively (Phiri *et al*. 2007) and those in Palestinian chickens, where the prevalence of *A. galli* and *H. gallinarum* was 75.6% and 68.9%, respectively (Rayyan & Al-Hindi 2010). In this study, variation in the prevalence of these parasites was observed between the two different provinces, although they were not statistically significant. Mukaratirwa and Khumalo (2010) observed more parasite species and relatively higher prevalence of *A. galli, H. gallinarum* and *Capillaria* spp. in coastal KwaZulu-Natal than was observed in this study. The significantly lower prevalence of *A. galli* and *R. cesticillus* parasites in KwaZulu-Natal and the relatively lower prevalence of the other nematodes and tapeworms were probably because of the generally dry summer months in the sampled localities of Msinga, in contrast to Limpopo Province where it rained during the days of sampling. In both provinces, all parasitised chickens harboured 1–6 helminth species, which was comparable to a study by Mukaratirwa and Khumalo (2010) but less than that from other studies that observed up to 7, 10 and 13 species of gastrointestinal helminths (Bersabeth 1999; Permin *et al*. 2002; Phiri *et al*. 2007). The level of mixed infestation observed in this study was expected and is common in village chickens (Phiri *et al*. 2007).

No trematodes were observed in the faecal samples and GIT of the village chickens in this study, which is in agreement with findings from previous studies in different populations (Abdelqader *et al*. 2008; Mukaratirwa & Khumalo 2010). However, this was in contrast with the study by Mwale and Masika (2011) who identified the trematodes *Postharmostomum gallium* and *Postharmostomum commutatum.* According to those authors, trematodes require a wide range of intermediate hosts such as dragonflies and freshwater snails to complete their life cycle, which may not be available in most production systems; therefore, they are rare.

## Conclusion

The study described the chicken production systems typical for small-holder village chicken farming in the Limpopo and KwaZulu-Natal provinces of South Africa. As observed in similar systems, village chickens contribute to the livelihood of the many families in marginalised communities of South Africa. The scavenging production system coupled with minimal management inputs and lack of knowledge on chicken health exposes the village chickens to different diseases and various internal and external parasites. Economically important parasites such as *A. galli* and *Eimeria* spp. were prevalent in both provinces, with variations in worm burdens and infection intensity. Overall, this study presents gastrointestinal parasites as a problem affecting the village chickens of Limpopo and KwaZulu-Natal provinces. Prevention and control of parasites in these farming systems are compromised by the mixed farming systems, the limited resources at the farmers’ disposal for chicken management and the absence of biosecurity measures to avoid disease and parasite transmission amongst chickens and interspecies.
